# Dual screen for gut metabolites suppressing enterobacterial growth and invasiveness reveals structure–activity relationships among anti-infective indoles

**DOI:** 10.1080/19490976.2026.2728235

**Published:** 2026-09-12

**Authors:** Alexandra Bergholtz, Weifeng Lin, Amanpreet Kaur, Anjeela Bhetwal, Shaochun Zhu, André Mateus, Maria Letizia Di Martino, Jens Eriksson, Daniel Globisch, Mikael E. Sellin

**Affiliations:** a Department of Medical Biochemistry and Microbiology, Uppsala University, Uppsala, Sweden; b Uppsala Antibiotic Center, Uppsala University, Uppsala, Sweden; c Department of Chemistry for Life Sciences, Uppsala University, Uppsala, Sweden; d Department of Chemistry, Umeå University, Umeå, Sweden; e The Laboratory for Molecular Infection Medicine Sweden (MIMS), Umeå, Sweden; f Science for Life Laboratory, Uppsala, Sweden

**Keywords:** Gut metabolites, metabolome, indole, methylindole, *Salmonella*, *Shigella*, epithelium, invasion, anti-infectives

## Abstract

The antibiotic resistance crisis has made the characterization of new anti-infective molecules a pressing matter. Molecules that suppress bacterial growth and survival, virulence, or a combination of these traits all warrant further exploration. Naturally occurring microbe‒host ecosystems, such as the human gut, provide incompletely tapped resources in this regard. We developed a flexible platform to parallelly assess how gut metabolites affect the growth and epithelial cell invasion capacity of the enteropathogens *Salmonella enterica* Typhimurium (*Salmonella*) and *Shigella flexneri*. By screening a gut metabolite library, the assays identified multiple anti-infective compound classes and extended previously reported antibacterial activities, e.g., for medium-chain fatty acids, bile acids, purine nucleotides, and indoles. Importantly, a targeted follow-up screen combined with chemical biology iterations showed how the anti-infective activity of indole is impacted by its derivatization. Specifically, a methyl group at either of the carbons of the indole scaffold potentiated its concentration-dependent suppressive effect on type-III-secretion-system virulence protein expression, flagellar motility (for *Salmonella*), and enterobacterial growth. By contrast, N1-methylation markedly attenuated the activity of indole and its C-derivatized versions. This study, hence, offers assays for dual growth and virulence analysis of invasive enterobacteria exposed to anti-infective candidate molecules, and informs on the structure–activity relationships among indole metabolites.

## Introduction

Pathogenic bacteria use their virulence factors to colonize host tissues, causing tissue destruction, inflammation, and potentially lethal systemic spread. Antibiotics remain our standard therapy for treating bacterial infections, but the spread of antimicrobial resistance mechanisms puts current treatment options at risk.^1^ In the search for new chemical agents that selectively suppress or disarm pathogens, while keeping microbe‒host ecosystems minimally altered, naturally occurring metabolites represent interesting starting points.^2^ Because of the staggering microbial and chemical complexity,^3^ the mammalian gut and its metabolome constitutes a particularly relevant resource for such exploration.

Aggressive enterobacteria such as *Salmonella enterica* serovar Typhimurium (*S*. Tm) and *Shigella flexneri* (*S. fl*) colonize the gut lumen and invade intestinal epithelial cells (IECs) by means of Type-3-Secretion-Systems (T3SS) that work in concert with species- and strain-specific accessory virulence factors.^4-7^ Enterobacterial virulence is costly and therefore tightly regulated in response to external cues.^8-13^ The resident gut microbiota provides colonization resistance against incoming pathogens by competition for space and nutrients,^14^ ^,^ ^15^ stimulation of host counter defenses,^16^ ^,^ ^17^ or production of microbial warfare molecules.^18^ ^,^ ^19^ In addition, gut metabolites produced by either microbiota or host have been shown to modulate bacterial virulence.^20^ Short-chain fatty acids (e.g. propionate and butyrate) reach high mM concentrations in the distal gut^21^ and suppress T3SS-1 expression in *S.* Tm,^22-24^ with virulence-modulating activities also ascribed to several medium- (MCFAs) and long-chain fatty acids (LCFAs).^25-29^ Bile acids,^30^ ^,^ ^31^ and the tryptophan-derived metabolite indole^32-35^ have similarly been linked to suppression of either enterobacterial virulence or growth. These molecules vary widely in abundance along the intestinal tract and throughout the feeding cycle, with estimates of ~0.7–86 mM bile acids in the duodenum of the fed state,^36^ and ~0.3–6.6 mM indole present in human fecal samples.^37^ Pathogen growth-suppressive activities have furthermore been recently assigned to adenine, adenosine,^38^ and their derivatives.^39-41^ These observations notwithstanding, the majority of the gut metabolome, and derivatives of major metabolite classes, remain to be functionally mined for anti-infective properties.

Towards this goal, we present an imaging-based screening platform to assess the impact of diverse gut metabolites on *S*. Tm and *S. fl* growth and IEC invasion capacity. Combining this information with a comprehensive and diverse library of singly-stocked gut metabolites revealed multiple metabolite classes capable of suppressing *S*. Tm invasiveness. A follow-up screen identified structural modifiers among indole derivatives, linking indole modification patterns to the anti-infective activity profile and potency. Most notably, we found that C-methylation at five positions (C2, C3, C5, C6 and C7) potentiated the suppressive effect of indole on enterobacterial T3SS expression, flagellar motility, and growth. By contrast, *N*-methylation at position 1 drastically attenuated these indole activities. Taken together, this study offers a new strategy to resolve anti-growth versus anti-virulence activities of metabolites present in the gut or elsewhere and provides insights into indole structure–activity relationships.

## Methods

### Ethics statement

Enteroids were established in prior studies,^42^ ^,^ ^43^ using human jejunal tissue resected during bariatric surgery. Enteroid culture and experimentation procedures were approved by the relevant governing body (Etikprövningsmyndigheten, Sweden) under license numbers 2010-157 (addenda 2010-157-1 and 2020-05754) and 2023-01524-01. Data presented in this study were derived from the enteroid culture Hu18-9jej.

#### Bacterial strains, plasmids, and culture conditions

The bacterial strains and reporter plasmids used in this study are listed in Table S1. As shown in Figure S3A–B, wild-type (*wt*) *Salmonella enterica* serovar Typhimurium (*S*. Tm) ATCC 14028 background was included.^44^ All other *S.* Tm strains were of SL1344 background (SB300, streptomycin resistant).^45^ Besides the *wt*, the previously characterized *ΔinvG* and *ΔmotA* strains were used.^42^ ^,^ ^46^ All *Shigella flexneri* (*S. fl*) strains were of the M90T background.^47^ Besides the *wt*, the previously described *ΔmxiD* strain was used.^48^ For infections in 96-well plate format, the indicated strain carried either the intracellular pM975 (p*ssaG*-GFPmut2) or pZ1400 (p*uhpT*-GFP) reporter plasmid.^49-52^ *S*. Tm strains were grown at 37 °C for 12 h in Luria Bertani broth (LB) with 0.3 M NaCl (Sigma-Aldrich) supplemented with appropriate antibiotics, on a rotating wheel (20 rpm). A 1:20 dilution was subcultured in fresh LB 0.3 M NaCl broth with or without the indicated metabolites or anti-infective compounds and grown for 4 h at 37 °C in either 96-well plates (Sarstedt #83.3924) on a rotating table (40 rpm), or in Falcon tubes on a rotating wheel (20 rpm), followed by dilution in cell culture medium for IEC infection. *S. fl* strains were grown at 30 °C for 16 h in LB supplemented with the appropriate antibiotics on a shaker (180  rpm) and subcultured 1:50 in fresh LB supplemented with or without metabolites or anti-infective compounds. The subcultures were grown in either 96-well plates at 30 °C for 15  min followed by 2 h 15  min at 37 °C on a rotating table (40 rpm), or in Falcon tubes on a shaker (180 rpm) at 37 °C for 2 h, and thereafter diluted in cell culture medium for IEC infection. The total volume of the subcultures in the 96-well plates was 100 µl.

#### Caco-2 cell culture

Caco-2 cells were cultured in DMEM GlutaMAX (Gibco) with 10% heat-inactivated fetal bovine serum (FBS; Gibco), 0.1  mM non-essential amino acids (NEAA; Gibco) and 1x PenStrep (Gibco), at 37 °C and 10% CO_2_. Caco-2 cell cultures were passaged every 2–3 d at a ratio of 1:4–1:8. Briefly, the cells were detached using Trypsin-EDTA (Gibco) for 10  min at 37 °C and then spun down at 250xg for 4  min. The cell pellet was resuspended in fresh culture medium and distributed in T-75 flasks.

#### Establishment of Caco-2 monolayers for infection

For infections in the 96-well plate format, trypsinized, resuspended Caco-2 cells were enumerated in a Bürker chamber and seeded at 1.43 × 10^5^ cells/cm^2^ (confluent monolayers, *S*. Tm infection) or 1.79 × 10^4^ cells/cm^2^ (sub-confluent monolayers, *S. fl* infection) in a black 96-well culture plate (ibidi #89626) 2 days prior to infection. The culture medium was refreshed 1 day post seeding. On the day of infection, the monolayers were washed once with DMEM containing GlutaMAX, and thereafter incubated in fresh medium without antibiotics for 2 h prior to the addition of the bacterial inoculum. All incubation steps were performed at 37 °C with 10% CO_2_, and infections were carried out in DMEM GlutaMAX/FBS/NEAA.

#### Human enteroid culture

The human jejunal enteroids and culture conditions used in the study were characterized extensively in previous studies.^42^ ^,^ ^43^ ^,^ ^53^ Enteroids were cultured in 75% Matrigel (Corning) domes overlaid with human organoid growth medium (hOGM; StemCell) with 1x PenStrep (Gibco) and incubated at 37 °C and 5% CO_2_. Enteroid cultures were passaged every 6–8 days at a ratio of 1:8. Washing was performed in DMEM/F12/0.25% BSA and included centrifugation at 300xg for 5 min at 4 °C. For splitting, enteroids were manually disrupted by pipetting in gentle cell dissociation reagent (StemCell), followed by washing. Fragments were resuspended in hOGM and Matrigel at a ratio of 1:3 and divided into 50 µl Matrigel domes in 24-well plates. The overlying hOGM medium was exchanged every 2‒4 days.

#### Establishment of human enteroid monolayers for infection

For infections in the 96-well plate format, enteroids were dissociated into single cells using TrypLE Express (Gibco) for 5‒10 min at 37 °C and then spun down at 300xg for 5 min at 4 °C. The cell pellet was washed once and subsequently resuspended in hOGM with 1x PenStrep and 10  µM Y-27632 (ROCK inhibitor, StemCell). The cells were enumerated in a Bürker chamber and seeded at 4.46 × 10^5^ cells/cm^2^ in a black 96-well culture plate (ibidi #89626) precoated with 40x diluted Matrigel. The ROCK inhibitor was omitted from the medium 2 days post-seeding. For an undifferentiated stem cell-like phenotype, monolayers were maintained in hOGM with 1x PenStrep until infection on day 3 post-seeding. For differentiation into an enterocyte-like phenotype, the hOGM medium was replaced with human enteroid differentiation medium (hODM; StemCell) with 1x PenStrep on day 3 post-seeding and maintained until infection on day 7. On the day of infection, the monolayers were washed once and thereafter incubated in fresh medium without antibiotics for 2 h prior to the addition of the inoculum. All incubation steps were performed at 37 C with 5% CO_2_, and infections were carried out in DMEM/F12.

#### Infection of intestinal epithelial cell layers with enterobacteria in a 96-well format

For inoculum preparation, reporter *S*. Tm subcultures were diluted to 2.0 × 10^8^ CFU per ml in the indicated cell culture medium, of which 50  µl was used to infect the IECs, resulting in 1 × 10^7^ CFUs per IEC monolayer. Where relevant, this also resulted in a 20-fold dilution of anti-infective compounds from the sub-cultivation step. Inocula of the indicated *S*. Tm strain were added atop the IEC monolayers, followed by incubation for 20  min (*S.* Tm SL1344) or 30  min (*S.* Tm 14028) to allow for bacterial invasion. The inoculum of reporter *S. fl* was prepared by diluting the subculture to 2.5 × 10^7^ CFU per ml in the indicated cell culture medium, of which 50  µl was used for infection, resulting in 1.25 × 10^6^ CFUs per IEC monolayer. Where relevant, the final concentration of the anti-infective compounds was 40-fold lower than that in the sub-cultivation step. After the addition of the *S. fl* inoculum to the monolayers, the plate was centrifuged at 700xg for 10 min prior to incubation for an additional 45 min to allow for bacterial invasion. Following IEC monolayer infections, IECs were washed two times and incubated in fresh medium supplemented with 100 µg/ml gentamicin until 3 h post infection to eliminate noninvaded bacteria. For infections with OD-corrected inocula, each subculture of *S*. Tm and *S. fl.* were first centrifuged for 5  min at 20,000 rpm and subsequently resuspended in a smaller fraction of spent medium in order to achieve an OD_600_ equal to that of the mock-treated control. Mock-treated controls were spun down and resuspended without adjusting the volume of the supernatant. Inocula were prepared from the OD-corrected subcultures and used for infection as described above. The optical density at 600 nm (OD_600_) of each subculture was measured using a Tecan Infinite M200 microplate reader. The background OD_600_ value from the LB-medium was subtracted from the subculture OD_600_. The relative OD_600_ was calculated as the OD_600_ for the indicated strain or treatment relative to *wt* or mock-treated control.

#### Platform for quantification of epithelial invasion by enterobacteria in 96-well format

Following IEC monolayer infections and elimination of non-invaded bacteria by gentamicin, monolayers were fixed in 2% paraformaldehyde for 20 min, washed twice with DPBS (Gibco), and stained with DAPI (1 µg/ml, Sigma Aldrich) and phalloidin-AlexaFluor647 (1 U/ml, Molecular probes) in 0.1% Triton X-100 for 30 min. After staining, the wells were imaged on a Nikon Ti2 microscope equipped with a 40x/0.6 NA Plan Apo air objective (pixel size 0.29 µm) and a back-lit sCMOS camera (Prime 95B, Photometrics). The fluorescence was excited using a Spectra-X light engine (Lumencor), with DAPI (390/22x), GFP (475/34x) and Alexa647 (ET640/30x). A quadruple bandpass dichroic and emission filter (Chroma 89402bs & 89402m) was used for DAPI, GFP and Alexa647 detection. Nine images per well in a 3 × 3 grid were automatically acquired using the µManager-high content screening plugin HCS Site generator.^54^ Images were analyzed with the open-source software CellProfiler^55^ in combination with the convolutional network Cellpose for segmentation and subsequent quantification of epithelial cells.^56^ Cellpose was chosen in favor of traditional segmentation algorithms owing to its superior performance during initial comparative testing. Briefly, flatfield correction images were generated according to previous work by Model and Burkhardt.^57^ IECs segmentation was performed on actin (Alexa647) and nuclear (DAPI) intensity images using the Cellpose cyto2 model. Invasion foci were detected using manually determined threshold values for pixel size and fluorescence intensity in the GFP channel with the CellPofiler IdentifyPrimary module. The threshold for fluorescence intensity was determined based on the background noise from non-infected control wells. The GFP fluorescence signals outside of the segmented IECs were removed using the MaskObject module. For *S*. Tm, the invasion ratio was calculated as the ratio between the number of invasion foci and the number of IECs per well. For *S. fl*, the invasion ratio was calculated as the ratio between the number of infected IECs and the number of IECs per well. The relative invasion was calculated as the invasion ratio for the indicated strain or treatment relative to *wt* or mock-treated control.

#### Human gut metabolite library screen

The compounds from the human gut metabolite library (MetaSci Inc., Ontario, SKU: MSIFEC0001) are listed in Table S2. Each compound was dissolved at 100  mM in DPBS (Gibco) supplemented with 5% DMSO and stored at −20 °C overnight. On the day of the experiment, the metabolite solutions were thawed for 30  min at room temperature, followed by centrifugation at 14,000 rpm for 1.5  min, and thereafter added to the 96-well plate together with *S*. Tm (final DMSO concentration of 0.5%). After 4  h of sub-cultivation, *S*. Tm inocula were prepared as described above and used to infect Caco-2 cell monolayers in the 96-well plate infection assay. Reference metabolites were initially screened across a range of concentrations, after which 10 mM was selected as the standard concentration for the main screen, with 1 mM concentration samples also included for internal reference. All screening experiments were performed in duplicate. Each plate assayed 22 different metabolites from the library, four mock-treated controls, two gentamicin-pretreated controls (100 µg/ml) and two uninfected control wells. Metabolites with two replicates suppressing *S*.Tm relative invasion ≤0.5 were classified as hits. The relative OD_600_ < 0.5 was used as a cut-off for anti-growth activity. A selection of hit metabolites was diluted in a 7-point 1:2 serial dilution series starting from the highest concentration used in the main screen and validated in the 96-well plate infection assay as described above.

#### Indole derivative screen

The indole derivatives screened in this study (Sigma-Aldrich, Sweden) are listed in Table S3. Each indole derivative was dissolved at 2.1 mM in LB 0.3 M NaCl supplemented with 0.5% DMSO and thereafter centrifuged at 14,000 rpm for 5 min on the day of the experiment. LB (0.3 M NaCl/0.5% DMSO) was used for the mock-treated control. *S*. Tm was sub-cultured in medium supplemented with or without the indicated indole derivative in a 96-well plate format as described above. After 4 h of sub-cultivation, *S*. Tm inocula were prepared as described above and used to infect Caco-2 cell monolayers in the 96-well plate infection assay. All indole derivatives were screened in an 8-point 1:2 serial dilution series starting from 2 mM. Each plate was assayed for four different indole-derivatives in duplicate, one internal dilution series of indole, 12 mock-treated controls, two gentamicin-pretreated controls (100 µg/ml) and two uninfected control wells.

#### Indole IC_50_ determination

The absolute inhibitory concentration of indole and indole derivatives required for 50% suppression of *S*. Tm invasion into Caco-2 cells (IC_50_) was calculated from dose‒response curves generated from the invasion ratio data obtained in the indole derivative screen. The 100% response was defined as the mean invasion ratio for mock-treated controls and the lowest concentration of indole-derivatives within the same experiment. The 0% response was defined as the mean invasion ratio obtained from high-dose gentamicin-treated controls (100 µg/ml). A non-linear curve fit was applied to the dose‒response curves using GraphPad Prism (settings: log(inhibitor) vs normalized response, standard slope: Hill slope = −1). The data are presented as the IC_50_ relative to the internal indole-treated control. Indole derivatives with <2 concentrations below the predicted 50% response were excluded from the IC_50_ determination.

#### Enterobacterial growth assay

*S*. Tm or *S. fl* subcultures were prepared in a 96-well plate format as described in the indole derivative screen. Measurements of the culture’s OD_600_ were recorded automatically every 15  min during 5 h 45  min (growth until late exponential phase) in a Tecan Spark 10 M Multimode Plate Reader. The 96-well plates were kept with closed lids, in a humidity chamber, at 37 °C, and with continuous shaking between the measurements. The background OD_600_ values from the medium were subtracted from the bacterial culture OD_600_. The area under the growth curve (AUC) was calculated in GraphPad Prism (10.4.0) (settings: baseline = 0). Data presented as the AUC relative to the mock-treated control.

#### Chemical synthesis

All reagents and solvents were purchased from Sigma-Aldrich or Fisher Scientific and were used without further purification. The solutions were concentrated *in vacuo* on a Heidolph rotary evaporator. Thin layer chromatography (TLC) was performed on silica gel 60 F-254 plates. Visualization of the developed chromatogram was performed using fluorescence quenching. Chromatographic purification of the products was accomplished using flash column chromatography on Merck silica gel 60 (40−63 μm). NMR spectra were recorded on an Agilent 400 MHz spectrometer (_1_H NMR: 400 MHz, _13_C NMR: 100 MHz). Chemical shifts are reported in parts per million (ppm) on the *δ* scale from an internal standard. The multiplicities are abbreviated as follows: s = singlet, d = doublet, t = triplet, q = quartet, m = multiplet. High-resolution mass spectra were acquired in positive mode with an ESI source at a mass range of *m/z* = 50–1200 using a Maxis II ETD Q-TOF mass spectrometer (Bruker Daltonics, Germany). The NMR spectra and further chemical synthesis details can be found in the Supplementary methods.

#### RNA extraction

*S*. Tm subcultures were grown in a 96-well plate format as described in the indole derivative screen. The 4 h subcultures from three wells were pooled together, spun down, and resuspended in TES buffer (10 mM Tris HCl pH 8, 1 mM EDTA, 150 mM NaCl). Total RNA was extracted from three independent overnight cultures using acid phenol (Sigma Aldrich). All centrifugation steps were performed at 14,000 rpm at 4 °C. Briefly, the samples were incubated with 10% SDS at 95 °C for 5 min and thereafter cooled on ice. The samples were incubated at 65 °C for 3 min, followed by the addition of 200 µl of acid phenol. The samples were incubated at 65 °C for an additional 10 min and vortexed every 2 min. The samples were centrifuged for 10 min, and the aqueous phase was transferred to a new tube and mixed with 180 µl of chloroform. The samples were incubated at 65 °C for 5 min, followed by 5 min of centrifugation. The aqueous phase was then transferred to a new tube before the addition of 375 µl of ice-cold 100% EtOH. The samples were incubated at −80 °C for at least 40  min and thereafter centrifuged for 30  min. The pellets were washed with ice-cold 70% EtOH, centrifuged for 10 min, air dried, and dissolved in sterile water. The extracted RNA concentration was measured with a Nanodrop, and the RNA quality was checked by agarose gel electrophoresis. Total RNA was subjected to DNaseI (Thermo Fisher) treated at 37 °C for 30  min. Samples with strong growth-inhibitory effects were excluded from the analysis.

#### Real-time quantitative PCR

The oligonucleotide primers used in this study are listed in Table S4. Total RNA was reverse transcribed with a high-capacity cDNA RT kit (Thermo Fisher) as described previously.^12^ ^,^ ^58^ Real-time quantitative PCR was performed with Maxima SYBR Green^2X^ (Thermo Fisher). The reactions were run on a CFX384 Real-Time Systems instrument (Bio-Rad). The data was analyzed with the Bio-Rad CFX Maestro Software. Transcript levels were calculated using the 2^-ΔΔCT^ method.^59^ The housekeeping gene *recA* was used for normalization. The data are presented as transcript levels relative to the mock-treated control.

#### Mass spectrometry-based proteomics

*S*. Tm subcultures were grown in the presence or absence of 0.25–2 mM of the indicated indole derivative for 4 h in Falcon tubes as described above. Cultures (1 ml) were spun down and washed once in PBS, followed by lysis in 50 µl of 2% SDS and boiling at 95 °C for 10 min. The samples (5 µg) were denatured with 2% SDS and 20 mM Tris (2-carboxyethyl) phosphine, and digested with a modified sp3 protocol^60^ as described previously.^61^ Briefly, the samples were added to a bead suspension (10  µg of Sera-Mag Speed Beads 4515-2105-050250 and 6515-2105-050250 in 10  µl 15 formic acid and 30 µl ethanol) and incubated with shaking for 15  min at room temperature. Thereafter, the beads were washed four times with 70% ethanol, and the proteins were digested overnight by adding 1.25 mM Tris (2-carboxyethyl) phosphine, 40 µl of 5 mM chloroacetamide, and 200 ng of trypsin in 100 mM HEPES pH 8.5. The peptides were then eluted from the beads and dried under vacuum. Peptides were labeled with TMTpro (Thermo Fisher Scientific), pooled, and thereafter desalted with solid-phase extraction, using a Waters OASIS HLB μElution Plate (30 μm). The samples were then fractionated into 48 fractions on a reversed-phase C18 system running under high pH conditions, with every sixth fraction being pooled together. Using a data-dependent acquisition strategy on a ThermoFisher Scientific Vanquish Neo LC coupled with a ThermoFisher Scientific Orbitrap Exploris 480, the samples were analyzed by liquid chromatography with tandem mass spectrometry. The raw files were processed with MSFragger^62^ (v.3.0) against the NCBI Salmonella Typhimurium SL1344 genome (FQ312003 and HE654724.1) using standard settings for TMT. Data from each TMT channel was normalized to the median of the run, and statistical significance was determined using limma (v.3.62.2).

#### Analysis of single-bacterium swimming speed

*S*. Tm subcultures were grown in the presence or absence of 1 mM of the indicated indole derivative in a 96-well plate format as described in the indole derivative screen. The 4 h subcultures were diluted 1:250 in fresh medium containing the corresponding indole-derivative and subsequently added to a black-walled glass-bottom 96-well plate (CellVis #P96-1.5H-*N*). Live DIC imaging was initiated 10 min after addition to the plate, on a Nikon Ti2 microscope using a 60x/0.7 S Plan Fluor air objective (pixel size 183 nm). Images were acquired at 250 ms intervals for 6 frames (1.25 s). The time-lapse series was imported to ImageJ and corrected for uneven background before single-bacterium swimming speed was tracked using the TrackMate plugin^63^ of ImageJ as previously described.^64^ Tracks with swimming speed < 5 µm/s (defined as non-motile) were excluded from the analysis.

#### Statistical analysis

Data were analyzed and plotted using R studio.^65^ The main packages used in R were tidyverse, ggbreak, multcomp, car and rstatix. Statistical analysis was performed as described in each figure legend. Statistical significance was determined using the functions aov() followed by glht() for one-way ANOVA and Dunnett’s post hoc test, or lm() followed by anova() for two-way ANOVA. A two sample t-test was performed using the function t.test() with the p.adjust() and “holm” method for multiple comparisons. The Kruskal-Wallis test was performed using the function kruskal.test() followed by dunn_test() and Bonferroni correction. Linear regression was performed using the lm() function.

## Results

### An assay system to quantify enterobacterial growth and IEC invasion following gut metabolite exposure

**Figure 1.**
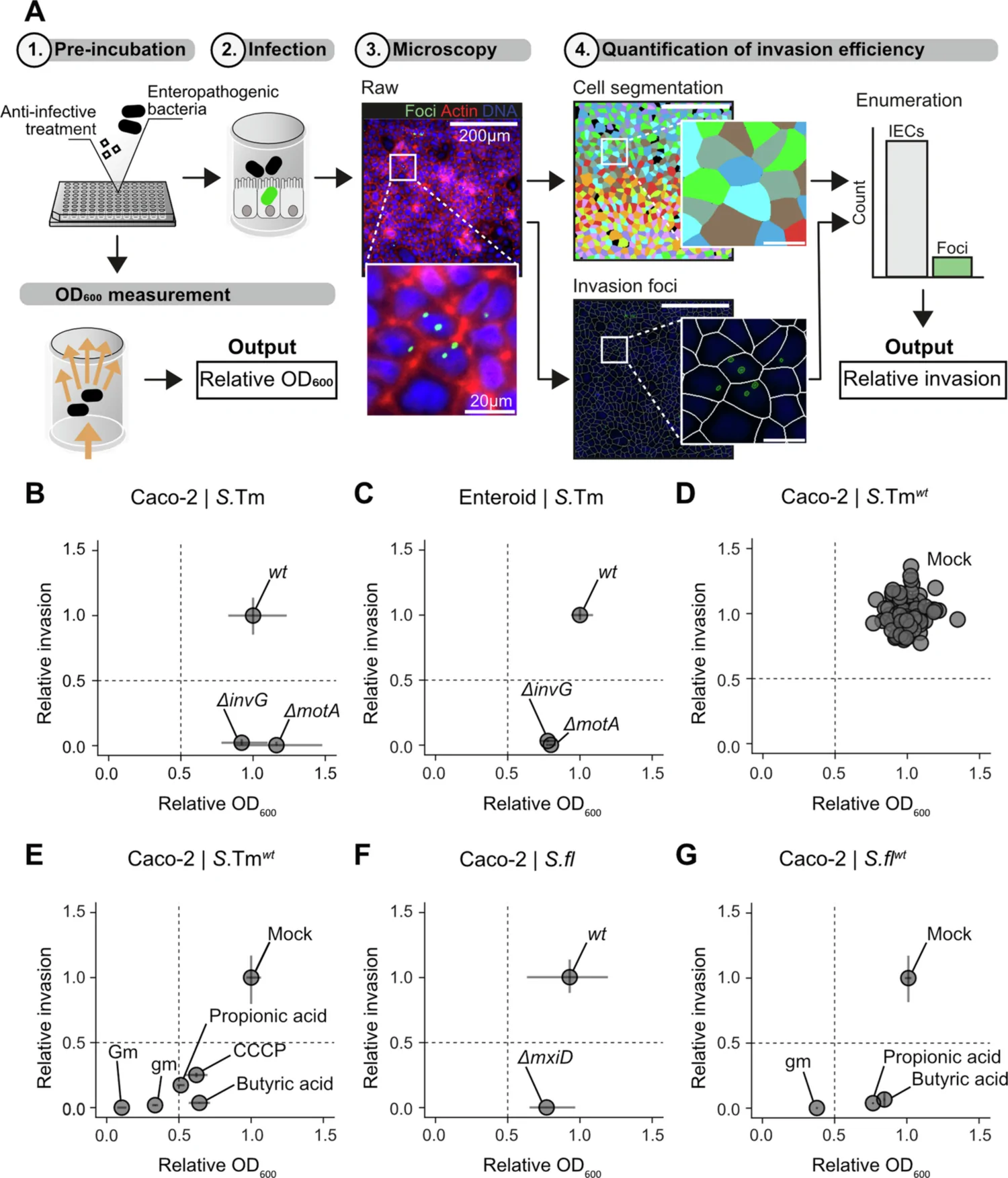
A 96-well format platform for dual quantification of enteropathogenic bacterial growth and epithelial cell invasion. (A) Schematic illustration of the 96-well format of the IEC monolayer infection assay: enteropathogenic bacteria carrying the intracellular GFP reporter construct p*ssaG*-GFP (*S*. Tm) or p*uhpT*-GFP (*S. fl)* were incubated in the presence or absence of metabolites/compounds of interest prior to infection of IECs and OD_600_ measurement. The infected IEC monolayers were fixed in 2% PFA, stained with DAPI (blue) and Alexa Fluor 647 Phalloidin (red), and imaged using semi-automated fluorescence microscopy. IECs and invasion foci are enumerated using the image analysis software CellProfiler in combination with the cell segmentation algorithm Cellpose. The obtained OD_600_ and invasion ratio for each infection were normalized to their corresponding *wt* or mock-treated control (“relative OD_600_” and “relative invasion”). See Methods for further details. (B–G) *S*. Tm or *S. fl* relative OD_600_ (x-axis) and relative invasion into IECs (y-axis). (B) Caco-2 cell monolayers infected with T3SS-1-deficient *S.* Tm^*ΔinvG*^ (*n* = 28), non-motile *S*. Tm^*ΔmotA*^ (*n* = 32) and *S*. Tm^*wt*^ (*n* = 32). (C) Undifferentiated human enteroid-derived monolayers infected with T3SS-1-deficient *S*. Tm^*ΔinvG*^ (*n* = 2), non-motile *S*. Tm^*ΔmotA*^(*n* = 2) and *S*. Tm^*wt*^ (*n* = 3). (D) Caco-2 cell monolayers infected with mock-treated *S*.Tm^*wt*^ (*n* = 91). Each dot represents the relative invasion ratio and relative OD_600_ from individual wells relative to the mean for all replicates. (E) Caco-2 cell monolayers infected with *S*. Tm^*wt*^ pre-incubated with anti-infective metabolites/compounds: butyric acid (10  mM), propionic acid (10  mM), CCCP (50  µM), high-dose gentamicin (Gm, 100 µg/ml), low dose gentamicin (gm, 10 µg/ml) (*n* = 2), and mock-treated control (*n* = 4). (F) Caco-2 cells infected with T3SS-deficient *S. fl* ^*ΔmxiD*^ (*n* = 6) and *S. fl* ^*wt*^ (*n* = 6) strains. (G) Caco-2 cells infected with *S. fl* ^*wt*^ pre-incubated with anti-infective metabolites/compounds: butyric acid (5  mM), propionic acid (5  mM), low-dose gentamicin (gm, 10 µg/ml) (*n* = 2), and mock-treated control (*n* = 3). The data plotted represent the mean with range. Dotted lines depict the 0.5 cut-offs.

To screen for gut metabolite effects on enterobacterial growth and virulence, we developed a 96-well-format assay to quantify *S*. Tm inoculum behavior and invasion of cultured epithelial cell layers (inspired by^66^). Human Caco-2 cells, or human enteroid-derived IECs, were seeded and matured into confluent monolayers. A *S*. Tm strain carrying the p*ssaG*-GFP reporter (which turns GFP+  when arriving in the *Salmonella*-containing vacuole following invasion)^49^ ^,^ ^50^ was prepared and subcultured for 4 h in the presence or absence of the tested metabolites. The subculture mixture was diluted 1:20 and used as an inoculum to infect the epithelial monolayers (20  min, MOI 50), followed by incubation in gentamicin-containing medium to eliminate extracellular bacteria and allow p*ssaG*-GFP reporter maturation. Infected monolayers were counterstained, followed by quantification of total IECs and intraepithelial *S*. Tm invasion foci (GFP + spots) by a semi-automated microscopy pipeline (Figure 1A, Figure S1A-C; see Methods). OD_600_ of the 4 h *S*. Tm subcultures were measured in parallel to quantify the effects on bacterial growth. For both scoring of *S*. Tm growth and invasion foci frequency, results were normalized to the corresponding data for unperturbed *S*. Tm *wt*/p*ssaG*-GFP, giving rise to the metrics “relative OD_600_” and “relative invasion” (Figure 1A; *wt* unperturbed = 1).

A set of control experiments was conducted to ensure robustness and resolution. The *S*. Tm T3SS-1 and flagella are well-characterized virulence factors known to drive IEC invasion.^4^ ^,^ ^67^ ^,^ ^68^ In line with this, *S*. Tm^*∆invG*^ (lacks a key T3SS-1 component;^46^) and *S*. Tm^*∆motA*^ (incapable of flagellar movement;^42^) reporter strains exhibited normal relative OD_600_, but dramatically reduced relative invasion in Caco-2 monolayers (Figure 1B, S1D; relative invasion 0.022 +/− 0.010 and 0.0037 +/− 0.0056, respectively). Similar results were noted for human enteroid-derived IEC monolayer infections (Figure 1C, S1E). However, the total number of invasion events was lower for infections of the enteroid-derived IEC monolayers, particularly following their maturation in an enterocyte differentiation medium (Figure S1B-C; in line with our recent study;^69^). This, combined with the propensity of non-transformed IECs to undergo swift cell death and extrusion upon *S*. Tm infection,^42^ ^,^ ^50^ ^,^ ^70^ makes the Caco-2 monolayers the more robust host cell model for the present application.

Any screen comes with the risk of false positives. To assess stochastic variability in the readouts, we carried out 91 parallel mock-infection assays in Caco-2, with *S*. Tm^*wt*^ reporter strain pretreated with only solvent (0.5% DMSO in PBS), and compared to the average of all the wells (Figure 1D). All 91 samples clustered around the mean (Figure 1D; mean ± sd for relative OD_600_ 0.957 +/− 0.092 and relative invasion 0.999 +/− 0.116). Based on this, conservative cut-off values of relative OD_600_ 0.5 and relative invasion of 0.5 were chosen, which should yield less than 1.1% (<1/91) false positives in a screening context. Applying those cut-offs, and using solvent (mock) treated *S*. Tm^*wt*^ as a reference, this assay system could readily detect the suppression of epithelial invasion by established gut metabolites and other chemical compounds (Figure 1E). Incubation with short-chain fatty acids (SCFAs; 10  mM) that interfere with *S*. Tm T3SS-1 virulence (propionic acid and butyric acid;^22-24^) as well as carbonyl cyanide *m*-chlorophenylhydrazone (CCCP; 50 µM) that nullifies the proton-motive force for flagellar motility,^71^ ^,^ ^72^ prior to infection (see Methods), reduced relative invasion (≤0.25). However, these factors had only modest effects on the relative OD_600_, which did not reach the cut-off value (Figure 1E). Further as anticipated, gentamicin (10–100 µg/ml) reduced the relative OD_600_ and consequently also decreased relative invasion in a dose-dependent manner. This finding illustrates that the assay system has a low inherent noise and can reconfirm the effects of known anti-infective metabolites and antibiotics on *S*. Tm growth and invasiveness.

Finally, we developed a similar assay for *S. fl*. The fluorescent reporter was exchanged for p*uhpT*-GFP (turns GFP +  upon bacterial arrival in the epithelial cell cytosol, the prevailing niche for *Shigellae*.^51^ ^,^ ^52^ ^,^ ^73^ The assay also included a spin step after inoculation to compensate for *Shigella'*s lack of motility. Similar to *S*. Tm, this assay system resolved differences in relative invasion between *S. fl* ^*wt*^ and *S. fl* ^*∆mxiD*^ (T3SS-deficient) strains (relative invasion of 0.0014 +/− 0.0013; Figure 1F, S1F). Moreover, short-chain fatty acids again suppressed relative invasion, while a low dose of gentamicin decreased the relative OD_600_, and consequently also relative invasion (Figure 1G).

### Screening of a gut metabolite library for anti-infective activities

**Figure 2.**
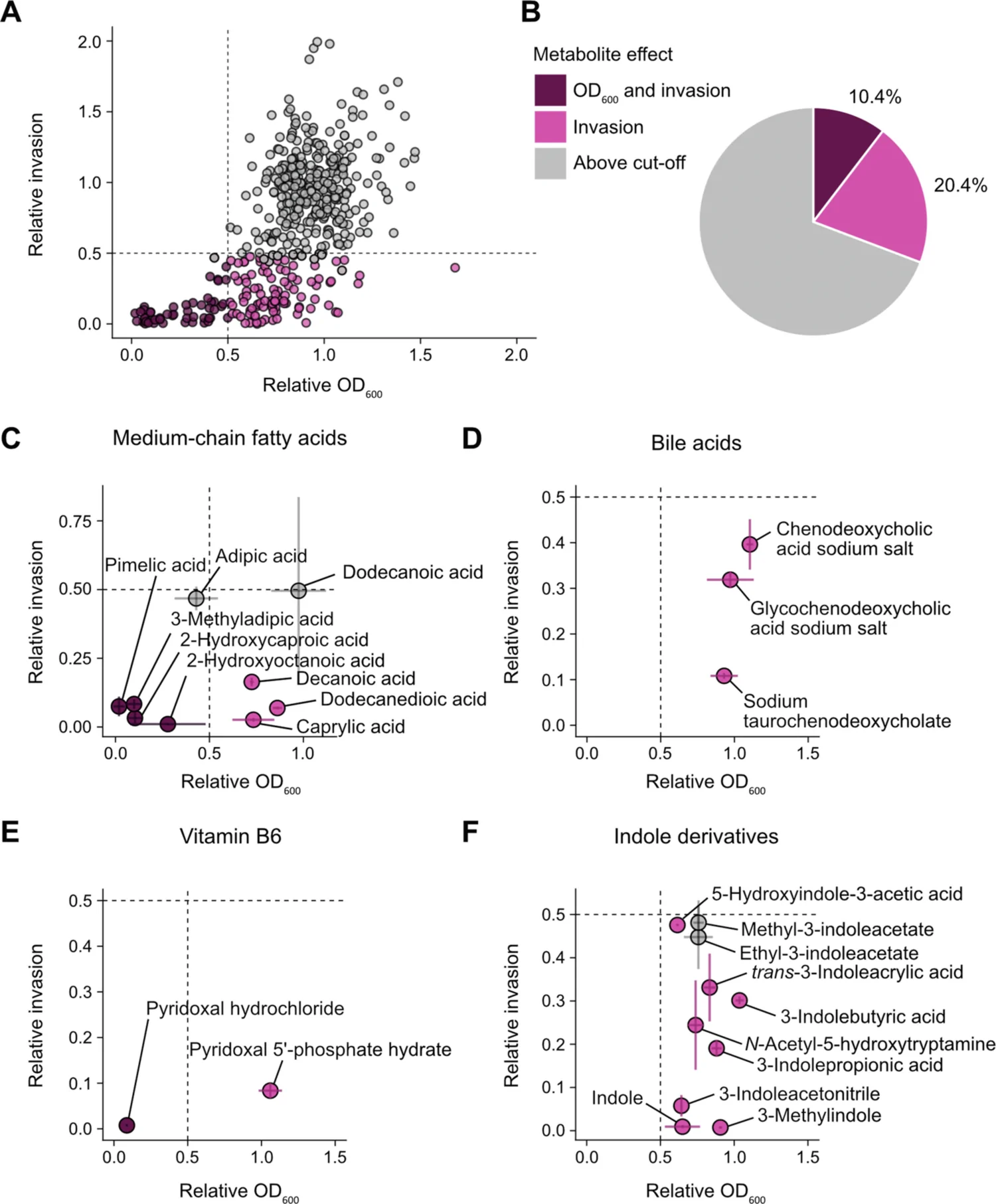
Screening of a gut metabolite library revealed functional classes and indole derivatives with variable anti-infective activity against *S*. Tm. (A) *S*. Tm relative OD_600_ and relative invasion into IEC (Caco-2) monolayers after individual 4 h pre-incubation with 481 metabolites from a human gut metabolite library. Each dot represents one metabolite result at 10  mM (*n* = 2 per condition). The fraction of metabolites with two replicates suppressing *S*. Tm relative invasion values ≤ 0.5 is classified as hits. The relative OD_600_ < 0.5 was used as a cut-off for anti-growth activity. (B) Categorization of metabolites from the library screen based on effect. The results are reported as percentages of the total number of screened metabolites. (C–F) *S*. Tm relative OD_600_ and relative invasion into IEC monolayers after 4 h pre-treatment with anti-infective metabolites from the library: (C) medium-chain fatty acids, (D) bile acids, (E) vitamin B6, and (F) indole derivatives. The data plotted represent the mean with range. The dotted lines depict the 0.5 cut-offs of relative OD_600_ (x-axis) and relative invasion (y-axis).

The *S*. Tm assay system was used to explore the anti-virulence and anti-growth properties of diverse human gut metabolites, implementing first an individually stocked library (MetaSci, SKU: MSIFEC0001; Table S2) with 481 compounds present in human fecal samples. The library comprises a variety of metabolites synthesized or modified by the gut microbiota, human biomolecules, and food breakdown products. The *S*. Tm^*wt*^ reporter strain was pre-incubated with each metabolite at a 10 mM concentration and the relative OD_600_ and relative invasion indices were assessed (as shown in Figure 1A), with mock-treated *S*. Tm^*wt*^ as a reference. Some metabolites exhibited signs of precipitation, predicted to cause an effective concentration <10  mM in those pre-incubation cultures. Nevertheless, applying the ≤0.5 cut-offs, 30.8% of all metabolites tested suppressed the bacterium´s relative invasiveness and/or growth (Figure 2A, B). Out of these, 98 (66.2% of “hits”) suppressed invasiveness (lower right quadrant in Figure 2A; relative OD_600_ ≥ 0.5, relative invasion ≤0.5), whereas 50 (33.8% of hits) suppressed both parameters (lower left quadrant in Figure 2A; relative OD_600_ < 0.5, relative invasion ≤0.5). Notably, not a single metabolite exclusively affected growth (upper left quadrant in Figure 2A). This makes sense, as any metabolite suppressing *S*. Tm growth during pre-incubation will, in this screening context, yield fewer input bacteria in the IEC invasion assay and hence also a lowered invasion index. These results demonstrate the robustness and limited noise of our assays.

The final activity list for the 148 “hit” metabolites was mined for common themes and functional compound classes. This resulted in the re-discovery of specific metabolites that have previously been linked to bacterial virulence suppression (e.g., caprylic acid^74^ ^,^ ^75^ and chenodeoxycholic acid^76^) (Figure 2C, D). Multiple medium-chain fatty acids (Figure 2C) and bile acid derivatives (Figure 2D), were among the metabolites that reduced *S*. Tm invasiveness, with four medium-chain fatty acids also impacting *S*. Tm growth at the present concentration (Figure 2C). Vitamin B6/Pyroxidal metabolites were also identified (Figure 2E), as well as purine nucleotides (Figure S2A). Moreover, 19 metabolites caused >1.5-fold higher relative invasion (Figure S2B), signifying a boosting effect on *S*. Tm virulence.

Among the metabolites suppressing *S*. Tm relative invasiveness below the ≤0.5 cut-off, no fewer than 10 indole derivatives were identified (Figure 2F). Indole itself is produced by gut microbiota members using tryptophan as a precursor, is found in a concentration range of ~0.3–6.6 mM in fecal samples, and has been assigned suppressive effects on bacterial virulence.^32-35^ ^,^ ^37^ ^,^ ^77^ Intriguingly, however, our results revealed a highly variable activity profile of indole derivatives with different side groups. For example, although both indole and short-chain fatty acids on their own suppress *S*. Tm virulence, the coupling of either a propionate or a butyrate side group to position 3 of indole resulted in weaker activity than for unmodified indole itself (Figure 2F). By contrast, 3-methylindole appeared highly potent. At the 10  mM screening concentration, both indole and 3-methylindole essentially abolished *S*. Tm invasiveness, with partial effects also on growth (Figure 2F). Lowering the concentrations resulted in dose-dependent partial suppression of *S*. Tm invasiveness and hinted at a higher potency for 3-methylindole than for indole (Figure S2C–D). These findings suggest the suitability of the assays to resolve structure–activity relationships among indole derivatives.

### A sub-screen of indoles provides structure–function insights and identifies a methylation position as a key anti-virulence determinant

**Figure 3.**
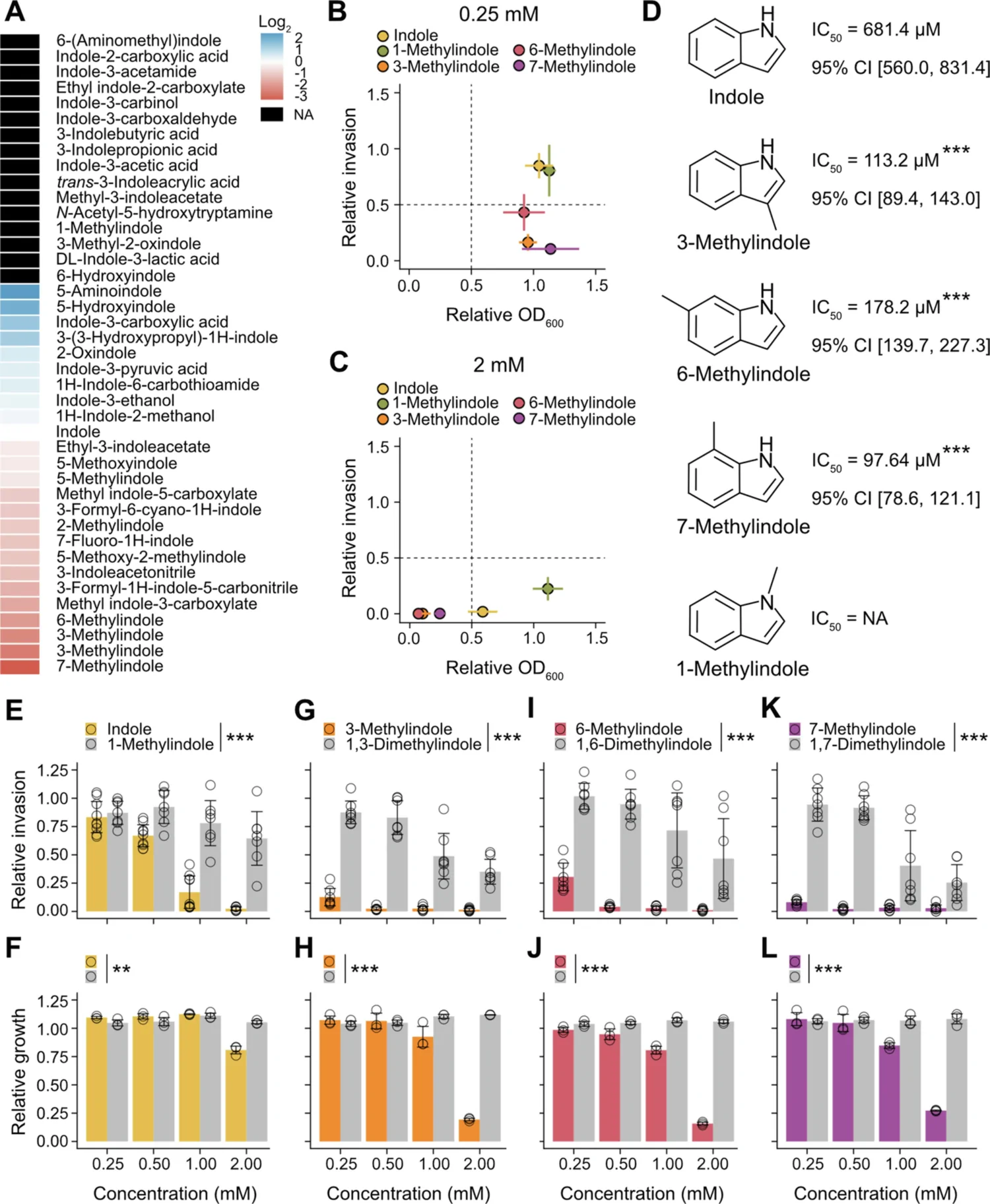
*C*-methylation of indole increases its anti-infective activity against *S*. Tm, while *N*-methylation attenuates it. (A) Absolute inhibitory concentration required for 50% suppression (IC_50_) of *S*. Tm invasion into IEC (Caco-2) monolayers for the indicated indole-derivatives reported as log_2_ relative to the indole derivative (*n* = 2). Indole derivatives that did not permit reliable IC_50_ determination (i.e., dose‒response curves with <2 concentrations below the 50% predicted response) were excluded from the analysis (black boxes). (B, C) *S*. Tm relative OD_600_ and relative invasion into IEC (Caco-2) monolayers after 4 h pre-treatment with indole (*n* = 12), 1-methylindole, 3-methylindole, 6-methylindole, or 7-methylindole (*n* = 8) at the concentration of (B) 0.25 mM and (C) 2 mM. The dotted lines depict the 0.5 cut-offs. D. Chemical structures and IC_50_ with 95% confidence interval for indole (*n* = 6), the top three most potent indole-derivatives; 3-methylindole, 6-methylindole, and 7-methylindole (*n* = 4). Chemical structure of 1-methylindole. NA, IC_50_ not determined (see Methods for rational). Statistical analyses via one-way ANOVA and Dunnett’s post hoc test, using IC_50_ for indole for comparison to *C*-methylated indoles. (E–L) *S*. Tm relative invasion into IEC (Caco-2) monolayers after 4 h pre-treatment with indole derivatives (methylated indoles, *n* = 7; indole, *n* = 9), and relative growth (area under the growth curve until the end of the exponential phase, *n* = 3) during sub-cultivation with indole derivatives. (E, F) Indole and 1-methylindole, (G, H) 3-methylindole and 1,3-dimethylindole, (I, J) 6-methylindole and 1,6-dimethylindole, or **(**K, L) 7-methylindole and 1,7-dimethylindole. Data plotted represent mean ± SD. Indole and *C*-methylated indoles were compared with their respective N1-methylated derivative by two-way ANOVA. **p < 0.01, ***p < 0.001. NA not applicable.

To explore substituents of the indole scaffold in relation to bioactivity, we conducted an additional sub-screen covering 40 indole derivatives (Table S3). These included both metabolites covered in the original screen and other naturally occurring and synthetic indole derivatives with functional side groups at various positions of the bicyclic indole scaffold (Figure 3A). Dose‒response curves (starting from 2 mM downwards) were generated for each compound, and the concentration required to suppress *S*. Tm IEC invasion by 50% (IC_50_) was calculated. The compounds were ranked by IC_50_ relative to unmodified indole, which under these conditions exhibited an absolute IC_50_ of 681.4  µM when averaged over 3 replicate experiments (Figure 3B–D). In total, 14 derivatives demonstrated higher potency (lower IC_50_), whereas nine derivatives showed lower potency (higher IC_50_) than unmodified indole (Figure 3A). The remaining 16 compounds were even less potent and did not permit reliable IC_50_ quantification (Figure 3A, an inhibitory effect beyond 50% for the two highest concentrations was not reached, hence prohibiting IC_50_ determination). Corroborating the original screening results and observations by others,^33^ we found that bulky substituents, such as the addition of a butyrate or a propionate moiety in position 3, lowered the indole potency (Figure 3A).

The hyperpotent indole compounds featured different side groups, but with a noticeable enrichment of methyl-indoles (Figure 3A). In addition to the previously identified 3-methylindole (included in duplicate from two sources), 2-, 5-, 6-, and 7-methylindole also showed stronger suppression of *S*. Tm invasiveness than indole (Figure 3A–C). The absolute IC_50_ values were broadly similar across these three derivatives: 113.2  µM (95% CI: 89.4–143.0 µM), 178.2  µM (139.7–227.3 µM), and 97.6  µM (78.6–121.1 µM) for 3-, 6-, and 7-methylindole, respectively (Figure 3D). By contrast, 1-methylindole (methylated at the amine of the pyrrole ring) was identified at the other end of the activity spectrum among compounds that did not qualify for IC_50_ determination (Figure 3A–D). A similar activity pattern was noted when employing another *S*. Tm wild-type strain (*S.* Tm 14028; Figure S3A, B). This suggests that *C*-methylation of the indole scaffold at essentially any position potentiates its suppressive effect on *S*. Tm invasiveness, while *N*-methylation at position 1 has the opposite effect.

Following these findings, we examined if N1-methylation would also reduce the effects of the hyperactive *C*-methylated indoles identified here. We therefore synthesized three di-methylated indole derivatives by methylation of the N1-position of 3-, 6-, and 7-methylindole, respectively (see Methods for synthesis protocol). The potency of purified 1,3-dimethylindole, 1,6-dimethylindole, and 1,7-dimethylindole (purity validated in Figure S4A–C) to suppress *S*. Tm growth and IEC invasion was compared to the respective monomethylated compounds. Synthetic N1-methylation of the indole scaffold itself abolished its suppressive effects (Figure 3E, F), in agreement with the results above (Figure 3A−D). Similarly, all three di-methylated indoles were dramatically attenuated compared to their 3-, 6-, or 7-mono-methylated counterparts (Figure 3G−L).

Of further note, indole and all C-mono-methylated derivatives consistently suppressed *S*. Tm invasion also at concentrations where growth appeared unaffected (compare the dose responses in Figure 3E, G, I, K with Figure 3F, H, J, L). By bacterial genetics, *S.* Tm growth inhibition effects were found to be uncoupled from the presence of a functional T3SS-1 (Figure S3C). Moreover, following normalization of the *S*. Tm inoculum density after treatment with higher (≥1 mM; growth-suppressive) indole/C-mono-methyl-indole concentrations, the suppressive effect on IEC invasion was still evident (Figure S3D, E). These findings altogether demonstrate (i) that a methyl group at either of multiple C-positions increases indoles anti-infective activity, (ii) that both indole and *C*-methylated indole derivatives suppress *S*. Tm invasiveness at low concentrations (<<1  mM) and *S*. Tm growth at higher concentrations (≥1 mM) and iii) that *N*-methylation at position 1 counteracts these activities across various indole derivatives.

### Methylation impacts indole derivative suppression of *Salmonella* virulence protein expression, motility, and enterobacterial invasivity

**Figure 4.**
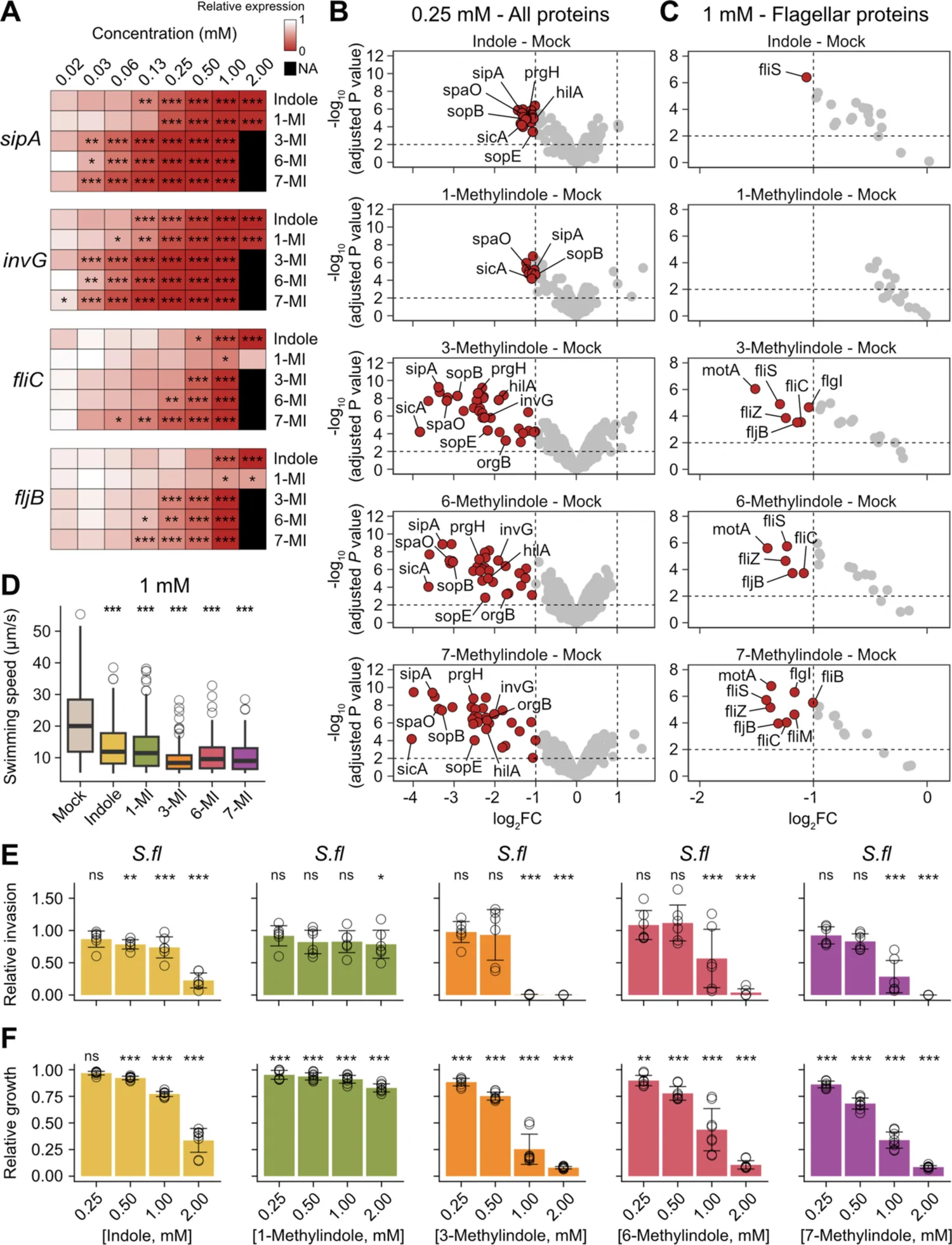
*C*-methylation of indole potentiates its suppressive effect on *S*. Tm virulence gene expression, flagellar swimming speed, and *S. fl* invasiveness. (A) Relative mRNA levels (2^-ΔΔCt^) of *S*. Tm virulence genes *invG*, *sipA*, *fliC,* and *fljB* after 4 h of cultivation with indole, 1-methylindole, 3-methylindole, 6-methylindole, or 7-methylindole relative to the mock-treated control (2^-ΔΔCt^ = 1; *n* = 3). Samples with strong growth-inhibitory effects were excluded from the analysis (black boxes). Statistical analyses via one-way ANOVA and Dunnett’s post hoc test were performed using mock-treated *S*. Tm (*n* = 32) for comparison to all other treatments. (B) Protein abundance in *S*. Tm after 4 h of cultivation with indole or the indicated methylated derivatives (as in A) at 0.25 mM. Annotation of selected T3SS-1-related proteins. (C) Flagellar protein abundance in *S*. Tm after 4 h of cultivation with indole or the indicated methylated derivatives (as in A) at 1 mM. (B, C) Each dot represents one protein. The differentially abundant proteins are shown in red, non-significant differentially abundant proteins are shown in gray. Significance was determined by limma: |log_2_FC|>1; adjusted *p-*value < 0.05. (*n* = 4). (D) Swimming speed atop glass of individual, motile *S*. Tm (defined as having a speed of > 5 µm/s, n ≥ 90 for each condition) cultured in the presence of 1 mM of the corresponding indole derivative for 4 h. The horizontal lines indicate the median, and the enclosing borders indicate the 25^th^ and 75^th^ percentiles. The length of the whiskers indicates 1.5 times the interquartile range. The open circles represent outliers. Statistical analyses via the Kruskal‒Wallis test, followed by Dunn’s test for multiple comparisons between mock- and indole-derivative-treated bacteria with Bonferroni comparison correction. (E) *S. fl* relative invasion into IEC (Caco-2) monolayers after 2.5 h pre-treatment with 0.25–2 mM indole, 1-methylindole, 3-methylindole, 6-methylindole or 7-methylindole (*n* = 6). (F) Relative growth (area under the growth curve until the end of the exponential phase) of *S. fl* cultivated with indole and methylated indole derivatives (as indicated in E; *n* = 8). (E, F) Statistical analyses via one-way ANOVA and Dunnett’s post hoc test, using mock-treated *S. fl* (relative invasion *n* = 30; relative growth *n* = 42) for comparison to all other treatment groups. The dose responses of the methylated indole derivatives were also compared to non-modified indole by two-way ANOVA (relative invasion/relative growth: 1-MI***/***; 3-MI**/***; 6-MI^ns^/***; 7-MI**/***; not shown in the figure panels). **p < 0.01, ***p < 0.001, ns not significant. NA not applicable.

Indole has been ascribed anti-infective properties against bacterial and fungal agents,^35^ ^,^ ^78-82^ including suppression of the expression and functionality of the virulence machinery.^32^ ^,^ ^33^ ^,^ ^82^ To probe the mechanistic basis for our observations, we employed quantitative RT-PCR to measure expression of representative *S*. Tm T3SS-1 (*invG*, *sipA*) and flagellar (*fliC*, *fljB*) genes (Figure 4A), and untargeted proteomics to probe global protein expression (Figure 4B, C, Figure S5; Table S5). In agreement with the phenotypic data (Figure 3), 3-, 6-, and 7-methylindole suppressed T3SS-1 and flagellar transcript levels to a greater extent than indole itself, with 1-methylindole showing reduced potency and causing essentially no reduction in flagellar transcripts (Figure 4A). Overall, the expression of T3SS-1 transcripts was affected at lower concentrations than that of flagellar transcripts for both indole and the three C-methylated indole derivatives (Figure 4A). Global untargeted proteomics substantiated these observations; among the 2311 identified proteins, T3SS-1-related proteins were selectively downregulated upon exposure to 0.25 mM indole, and their expression was markedly more reduced for 3-, 6-, or 7-methylindole (Figure 4B). Flagella-related proteins were also downregulated upon exposure to a relatively high concentration (1  mM) of 3-, 6-, or 7-methylindole, but with no effect of 1-methylindole (Figure 4C). In addition, the global proteomic changes instilled by 0.25  mM of the three hyperpotent C-methylindoles closely resembled the changes instilled by 1 mM indole (Figure S5A-C; R^2^ values of 0.8352–0.8985), suggesting a shared qualitative mechanism(s) with different potency.

We next monitored swimming dynamics in individual motile bacteria by differential interference contrast (DIC) time-lapse microscopy.^64^ This confirmed that 1 mM indole reduced the median swimming speed of still motile *S.* Tm (Figure 4D; 11.9 µm/s) relative to the mock-treated control (Figure 4D; 20.0 µm/s). Moreover, 3-, 6-, and 7-methylindole exhibited increased potency (Figure 4D; 8.3, 9.6, and 9.0, respectively), while no improved effect was observed for 1-methylindole (Figure 4D). Taken together, this demonstrates that the methylation pattern is a key determinant of the potency of indole as an anti-*S*.Tm agent, targeting multiple traits involved in bacterial growth, T3SS-1 machinery expression, and flagellar functionality.

Finally, we aimed to identify if the methylation-position-dependent augmentation/attenuation of indole activity generalizes beyond *S*. Tm. We assessed the impact of the compound series on *S. fl* growth and invasivity, using the adapted assay system (see Figure 1A, F, G). In contrast to *S*. Tm, *S. fl* can (for some strains) synthesize indole endogenously and also lacks flagellar motility.^83^ ^,^ ^84^ We found that the overall sensitivity to indole derivatives was lower for *S. fl* than for *S*. Tm (Figure 4E top panels, compared with Figure 3E, G, I, K). Nevertheless, both the relative IEC invasion and the relative growth of *S. fl* were suppressed by indole at concentrations of ~2 mM (Figure 4E, F). Importantly, 3-, 6-, and 7-methylindole were still hyperpotent, with detectable effects at 1  mM (Figure 4E, F), but 1-methylated indole had a negligible effect on *S. fl* invasivity and growth across the 0.25–2 mM concentration span (Figure 4E, F). In full agreement with the analogous *S.* Tm experiments above, indole/C-methylindole suppression of *S. fl* invasion and growth could be uncoupled by either inoculum density normalization or genetic T3SS ablation (Figure S6A–C). In summary, we conclude that *C*-methylation of indole at a variety of positions broadly potentiates its anti-enterobacterial effect on both virulence and growth, whereas N1-methylation has the opposite outcome.

## Discussion

The rapid spread of antimicrobial resistance (AMR) poses one of this century’s leading threats to human and animal health.^1^ An improved understanding of molecules that target microbial growth or virulence is vital to better understand these processes and to counter the threat of AMR. Although several classes of microbiota-derived metabolites with anti-virulence activity have been identified,^20^ the gut metabolome remains an incompletely explored resource.^85^ ^,^ ^86^ Importantly, traditional methods for assessing antimicrobial activity, e.g., broth microdilution and disc diffusion assays, do not detect anti-virulence activities, which emphasizes the need for developing tools to dissect the growth-suppressing versus virulence-suppressing activities of candidate molecules. To that end, we here established an imaging-based screening platform for parallel quantification of metabolite effects on bacterial growth and IEC invasion – a central virulence trait among Gram-negative enterobacteria.

Among the 481 gut metabolites initially screened for their anti-infective activity, our assays reconfirmed multiple classes of metabolites previously reported to influence *S*. Tm virulence, including MCFAs,^75^ LCFAs,^25^ ^,^ ^26^ bile acids,^30^ ^,^ ^31^ ^,^ ^76^ vitamin B6^87^ and purine nucleotides.^88^ The screen also re-identified indole,^77^ and an additional 9 indole derivatives, with variable anti-virulence potency against *S*. Tm. Extending on these findings, a sub-screen of 40 indole derivatives revealed that the composition and position of the side groups heavily impacted the anti-growth and anti-virulence properties of indole, hence expanding our understanding of the structure‒activity relationships within this molecule class.^89-93^ Notably, a C-methyl modification (to, e.g., positions 2, 3, 5, 6, and 7) enhanced both the anti-virulence and anti-growth activity of indole against *S*. Tm and *S. fl*, while the addition of a methyl group to N1 rendered the molecule dramatically less potent in suppressing IEC invasion and essentially eliminated its growth-inhibitory effect. The suggested importance of the N1-position chemical composition was further confirmed by *N*-methylation of the hyper-active *C*-methylated indoles, which in all cases led to loss of anti-infective activity. By contrast, a recent screen of indole derivatives against *C. parvum* demonstrated that parasite growth was inhibited by most derivatives regardless of substituent modification.^93^ However, that study investigated effects on a eukaryotic parasite in the infected host cell context, and did not include indoles substituted on the N1-position.

Indole has been shown to efficiently interact with and traverse membranes,^94^ ^,^ ^95^ and to act as a protonophore, dissipating the proton motive force (PMF) by facilitating H + flux across the cytoplasmic bacterial membrane.^96^ ^,^ ^97^ *C*-methylation will increase the overall lipophilicity (logP 2.29 for indole and 2.58–2.59 for 3-/6-/7-methylindoles by ALOGPS 2.1 prediction).^98^ ^,^ ^99^ We found essentially identical effects of a *C*-methyl group at various positions, and moreover, that *C*-methylated indoles induce similar phenotypic and proteomic changes as indole itself, but require lower concentrations to do so. Hence, the potentiation of activities to destabilize membranes and interfere with energy utilization provides a plausible explanation for why C2-, 3-, 5-, 6-, and 7-methylation augments the suppressive effects of indole on *S*. Tm and *S. fl*. Conversely, we propose that position N1-methylation, while also predicted to increase the overall lipophilicity, will make indole poor at acting as a protonophore due to the disruption of the NH-group at this position.

Membrane damage and/or dissipation of the PMF can, in a straight-forward way, explain suppressive effects on bacterial growth and flagellar motility. However, at lower concentrations than those needed for *S*. Tm growth inhibition, indole and its C-methylated derivatives still suppressed virulence transcript and protein expression, particularly for the T3SS-1 machinery. Moreover, even *N*-methylated indole (that had essentially no growth-inhibitory effect) was capable of suppressing *S*. Tm T3SS-1 transcript and protein expression to some extent. These observations point to additional indole activities impinging on virulence gene expression circuits and motility regulation, which gains support in recent literature.^32-34^ ^,^ ^100^ To resolve the multiple actions of indole derivatives on the deeper molecular level, how they can be tweaked by defined *C*- or *N*-position substitutions, and the extent to which stress‒response modules (e.g., PhoPQ and CpxAR;^33^ ^,^ ^34^ are involved in the effects, constitute exciting avenues for future research.

In summary, this study offers a platform for flexible dissection of anti-growth versus anti-virulence activities of metabolites and synthetic compounds that target enterobacteria. Moreover, our work provides foundational structure – activity insights that will facilitate the advancement of indole derivatives as research tools, or as scaffolds for future anti-infective development.

## Supplementary Material

Supplemental Material FigS1_07_Sep_2026_09_56_AU.tif

Supplemental Material Supplemental Material

Supplemental Material Supplemental Material

Supplemental Material Supplemental Material

Supplemental Material Supplemental Material

Supplemental Material Supplemental Material

Supplementary_methods Supplementary Methods

Supplemental Table Supplemental Table

Supplemental Table Supplemental Table

Supplemental Table Supplemental Table

Supplemental Table Supplemental Table

Supplemental Table Supplemental Table

## Data Availability

All data are presented in the figures and supplemental material. Original raw data files can be obtained from the corresponding authors upon reasonable request.

## Data deposition

The mass spectrometry proteomics data have been deposited to the ProteomeXchange Consortium via the PRIDE partner repository, dataset identifier PXD079402.
