# Supplementary material for: Dual screen for gut metabolites suppressing enterobacterial growth and invasiveness reveals structure–activity relationships among anti-infective indoles
Source: Gut Microbes. 2026 Sep 12;18(1):2728235. doi: 10.1080/19490976.2026.2728235 (PMC13577278; doi:10.1080/19490976.2026.2728235)
Supplement: Supplementary file 1 [file KGMI_A_2728235_SM2166.pdf]

Supplementary chemical synthesis

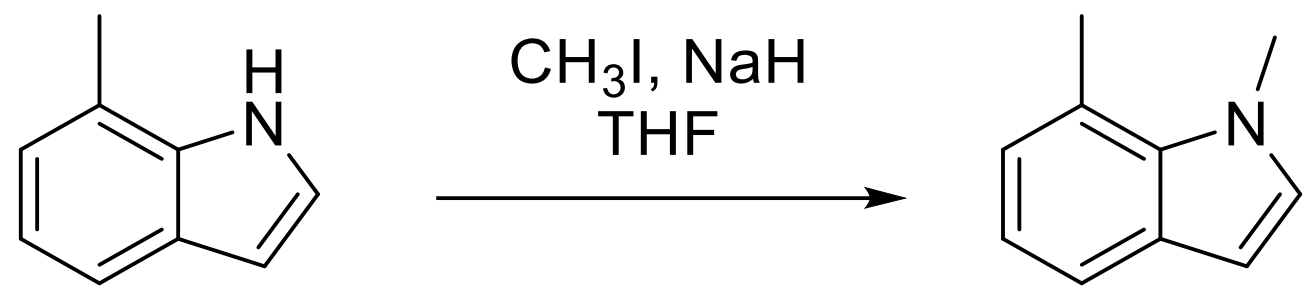

Synthesis of 1,7-dimethylindole

Methylation of indole was done according to the literature procedure(1). To a solution of indole (50 mg) in THF (30 mL) at 0 °C was added NaH (13.7 mg, 60 % dispersion in mineral oil). The heterogeneous mixture was stirred at 0 °C for 15 minutes and 1h at room temperature. The mixture was then cooled to 0 °C, treated with iodomethane (31 µL), and allow to warm to room temperature. After 30 minutes, the reaction mixture was cooled to 0 °C, quenched with saturated NH_4Cl (50 mL), and extracted with ether (3 x 30 mL). The organic layers were combined, washed with brine, dried over anhydrous Na_2SO_4, and concentrated in vacuo. The resulting oil was purified by flash chromatography (eluent: Hexane/EtOAc 10:1) to provide 1,7-dimethylindole (36.4 mg, 65.8%) as a colorless oil.

^{1}H NMR (400 MHz, $cdcl_3$) δ 7.49 (d, *J* = 7.8 Hz, 1H), 7.00 (t, *J* = 7.5 Hz, 1H), 6.96 (d, *J* = 3.1 Hz, 1H), 6.94 (d, *J* = 7.1 Hz, 1H), 6.47 (d, *J* = 3.1 Hz, 1H), 4.08 (s, 3H), 2.80 (s, 3H).

HRMS (ESI) *m/z* $[M+H]^+$ calcd for $C_{10}H_{12}N^+$ 146.0964; Found: 146.0965.

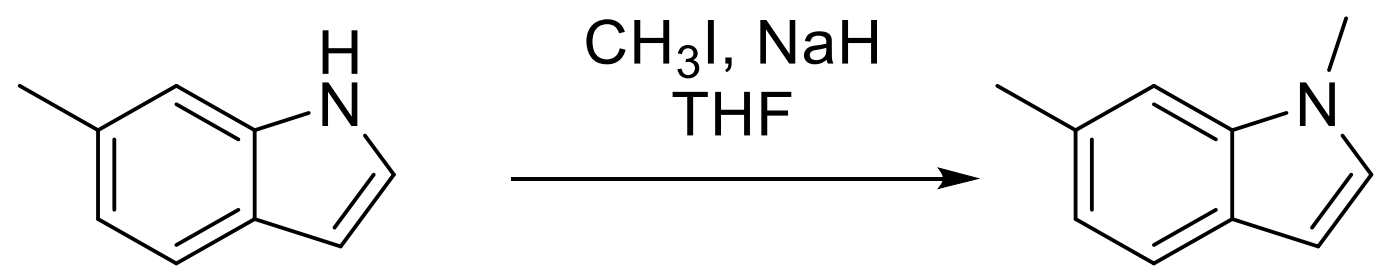

Synthesis of 1,6-dimethylindole

Methylation of indole was done according to the literature procedure(1). To a solution of indole (50 mg) in THF (30 mL) at 0 °C was added NaH (13.7 mg, 60 % dispersion in mineral oil). The heterogeneous mixture was stirred at 0 °C for 15 minutes and 1h at room temperature. The mixture was then cooled to 0 °C, treated with iodomethane (31 µL), and allow to warm to room temperature. After 30 minutes, the reaction mixture was cooled to 0 °C, quenched with saturated NH_4Cl (50 mL), and extracted with ether (3 x 30 mL). The organic layers were combined, washed with brine, dried over anhydrous Na_2SO_4, and concentrated in vacuo. The resulting oil was purified by flash chromatography (eluent: Hexane/EtOAc 10:1) to provide 1,6-dimethylindole (36.7 mg, 66.3%) as a colorless oil.

^{1}H NMR (400 MHz, $cdcl_3$) δ 7.56 (d, *J* = 8.0 Hz, 1H), 7.17 (s, 1H), 7.04 – 6.96 (m, 2H), 6.48 (d, *J* = 3.1 Hz, 1H), 3.78 (s, 3H), 2.55 (s, 3H).

HRMS (ESI) *m/z* $[M+H]^+$ calcd for $C_{10}H_{12}N^+$ 146.0964; Found: 146.0964.

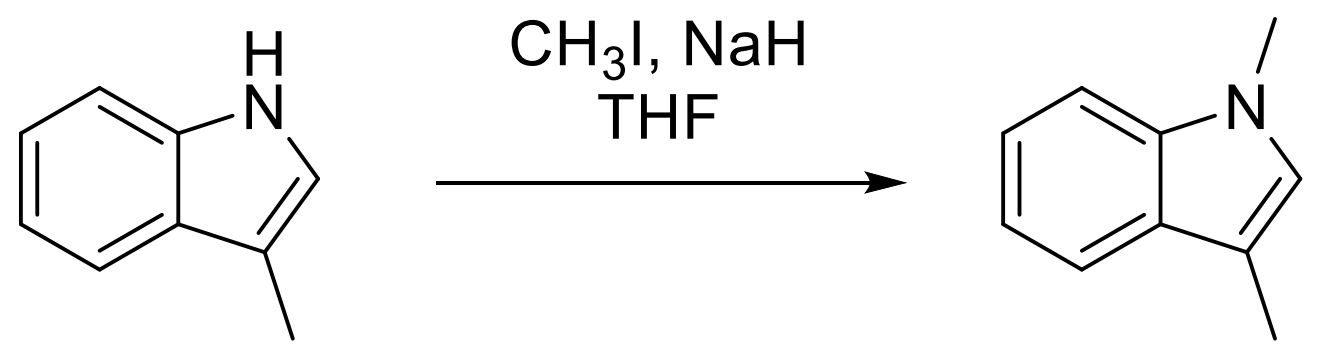

Synthesis of 1,3-dimethylindole

Methylation of indole was done according to the literature procedure(1). To a solution of indole (50 mg) in THF (30 mL) at 0 °C was added NaH (13.7 mg, 60 % dispersion in mineral oil). The heterogeneous mixture was stirred at 0 °C for 15 minutes and 1h at room temperature. The mixture was then cooled to 0 °C, treated with iodomethane (31 µL), and allow to warm to room temperature. After 30 minutes, the reaction mixture was cooled to 0 °C, quenched with saturated NH_4Cl (50 mL), and extracted with ether (3 x 30 mL). The organic layers were combined, washed with brine, dried over anhydrous Na_2SO_4, and concentrated in vacuo. The resulting oil was purified by flash chromatography (eluent: Hexane/EtOAc 10:1) to provide 1,3-dimethylindole (10.1 mg, 18.2%) as a colorless oil.

^{1}H NMR (400 MHz, cdcl$_3$) δ 7.56 (d, *J* = 7.9 Hz, 1H), 7.26 (t, *J* = 9.2 Hz, 1H), 7.21 (t, *J* = 7.5 Hz, 1H), 7.10 (t, *J* = 7.4 Hz, 1H), 6.81 (d, *J* = 1.3 Hz, 1H), 3.72 (s, 3H), 2.32 (s, 3H), 2.32 (d, *J* = 1.4 Hz, 3H).

HRMS (ESI) *m/z* $[M+H]^+$ calcd for $C_{10}H_{12}N^+$ 146.0964; Found: 146.0966.

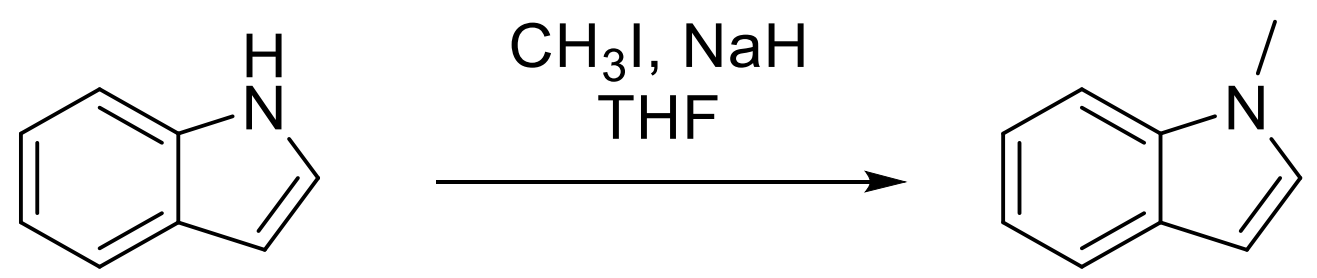

Synthesis of 1-methylindole

Methylation of indole was done according to the literature procedure(1). To a solution of indole (50 mg) in THF (30 mL) at 0 °C was added NaH (13.7 mg, 60 % dispersion in mineral oil). The heterogeneous mixture was stirred at 0 °C for 15 minutes and 1h at room temperature. The mixture was then cooled to 0 °C, treated with iodomethane (31 µL), and allow to warm to room temperature. After 30 minutes, the reaction mixture was cooled to 0 °C, quenched with saturated NH_4Cl (50 mL), and extracted with ether (3 x 30 mL). The organic layers were combined, washed with brine, dried over anhydrous Na_2SO_4, and concentrated in vacuo. The resulting oil was purified by flash chromatography (eluent: Hexane/EtOAc 10:1) to provide 1-methylindole (14.7 mg, 26.3%) as a colorless oil.

^{1}H NMR (400 MHz, cdcl$_3$) δ 7.65 (d, *J* = 7.8 Hz, 1H), 7.37 – 7.32 (m, 1H), 7.27 – 7.21 (m, 1H), 7.15 – 7.10 (m, 1H), 7.07 (d, *J* = 3.1 Hz, 1H), 6.50 (dd, *J* = 3.1, 0.9 Hz, 1H), 3.81 (s, 3H).

HRMS (ESI) *m/z* $[M+H]^+$ calcd for $C_9H_{10}N^+$ 132.0808; Found: 132.0810.

References Supplementary chemical synthesis

1. Xu XH, Liu GK, Azuma A, Tokunaga E, Shibata N. Synthesis of Indole and Biindolyl Triflones: Trifluoromethanesulfonylation of Indoles with Tf2O/TTBP (2,4,6-tri-tert-butylpyridine) System. Org Lett. 2011 Sep 16;13(18):4854–7. doi:10.1021/ol201931x

Supplementary NMR Spectra

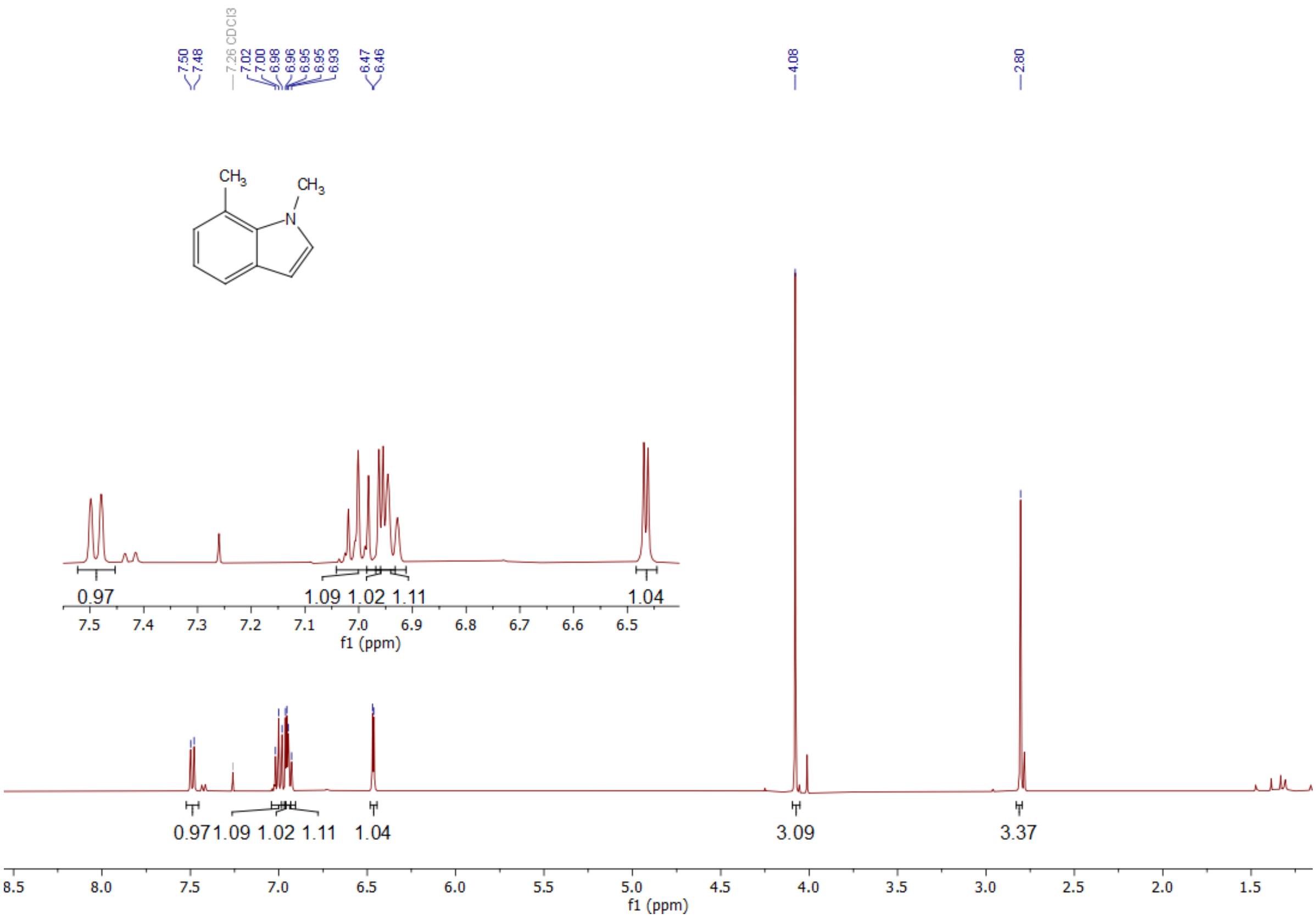

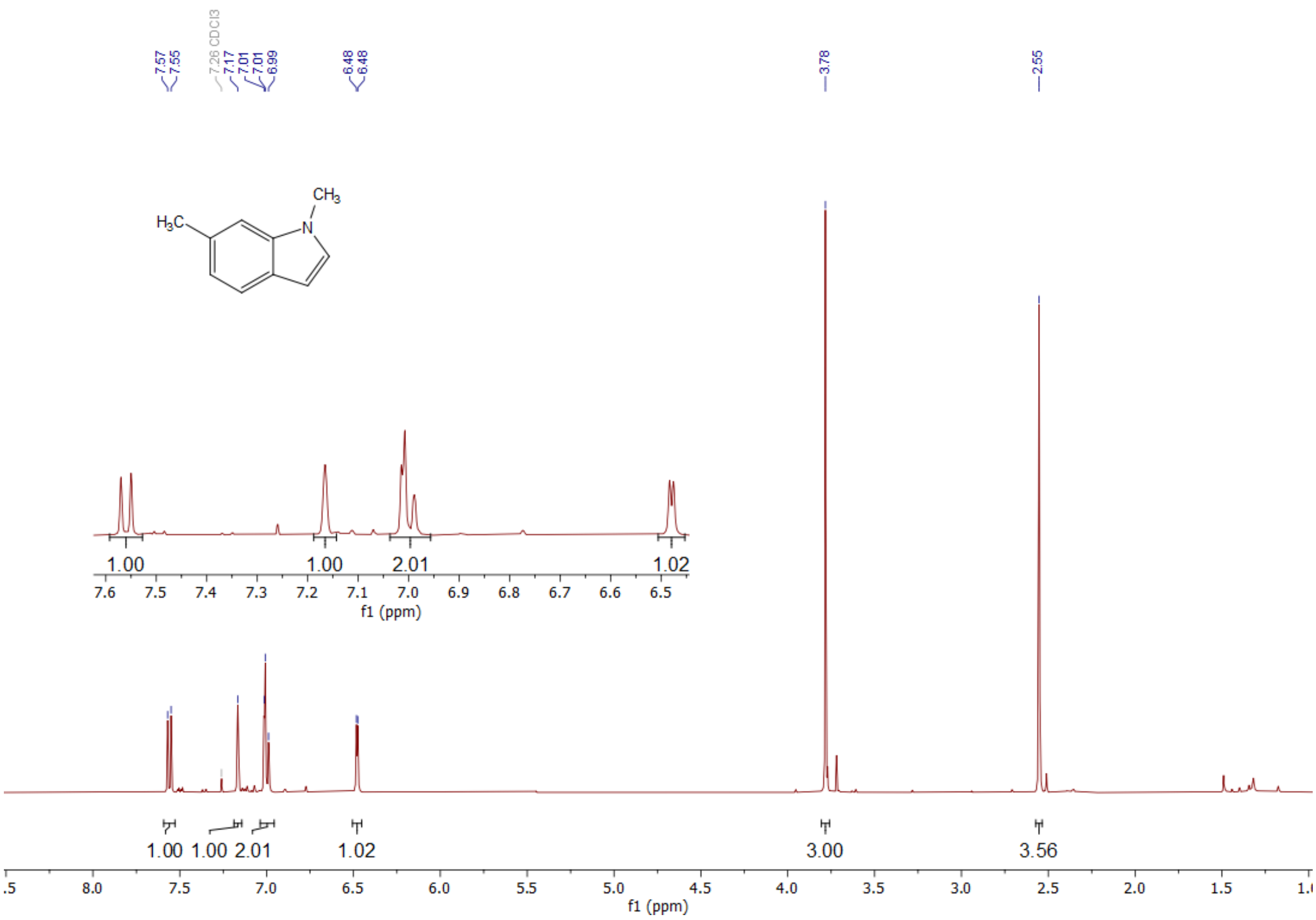

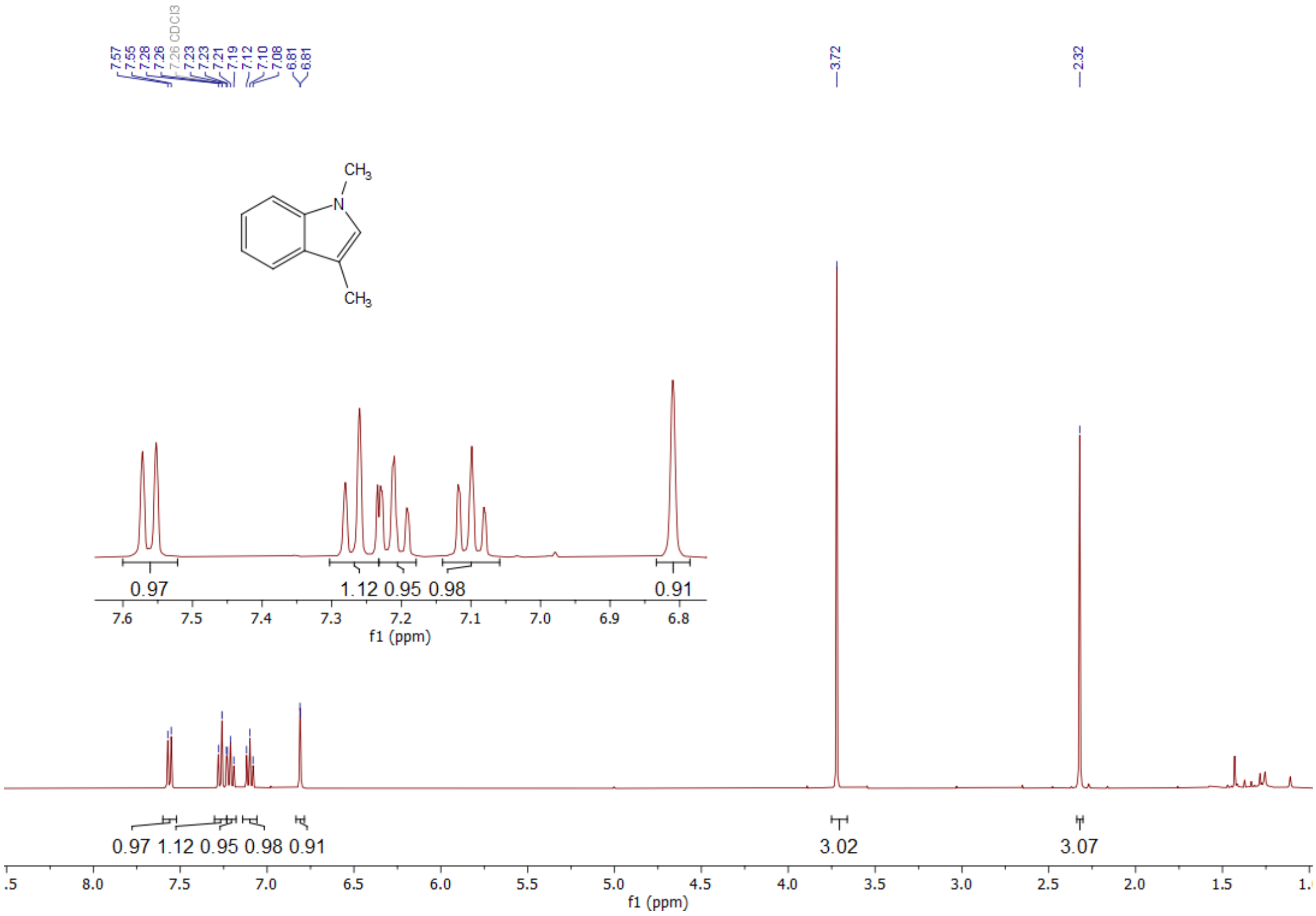
7.57
7.55
7.28
7.26 CDCl3
7.23
7.23
7.21
7.19
7.12
7.10
7.08
6.81
6.81
3.72
2.32
CH3
N
CH3
0.97
1.12 0.95 0.98
0.91
f1 (ppm)
0.97 1.12 0.95 0.98 0.91
3.02
3.07
f1 (ppm)

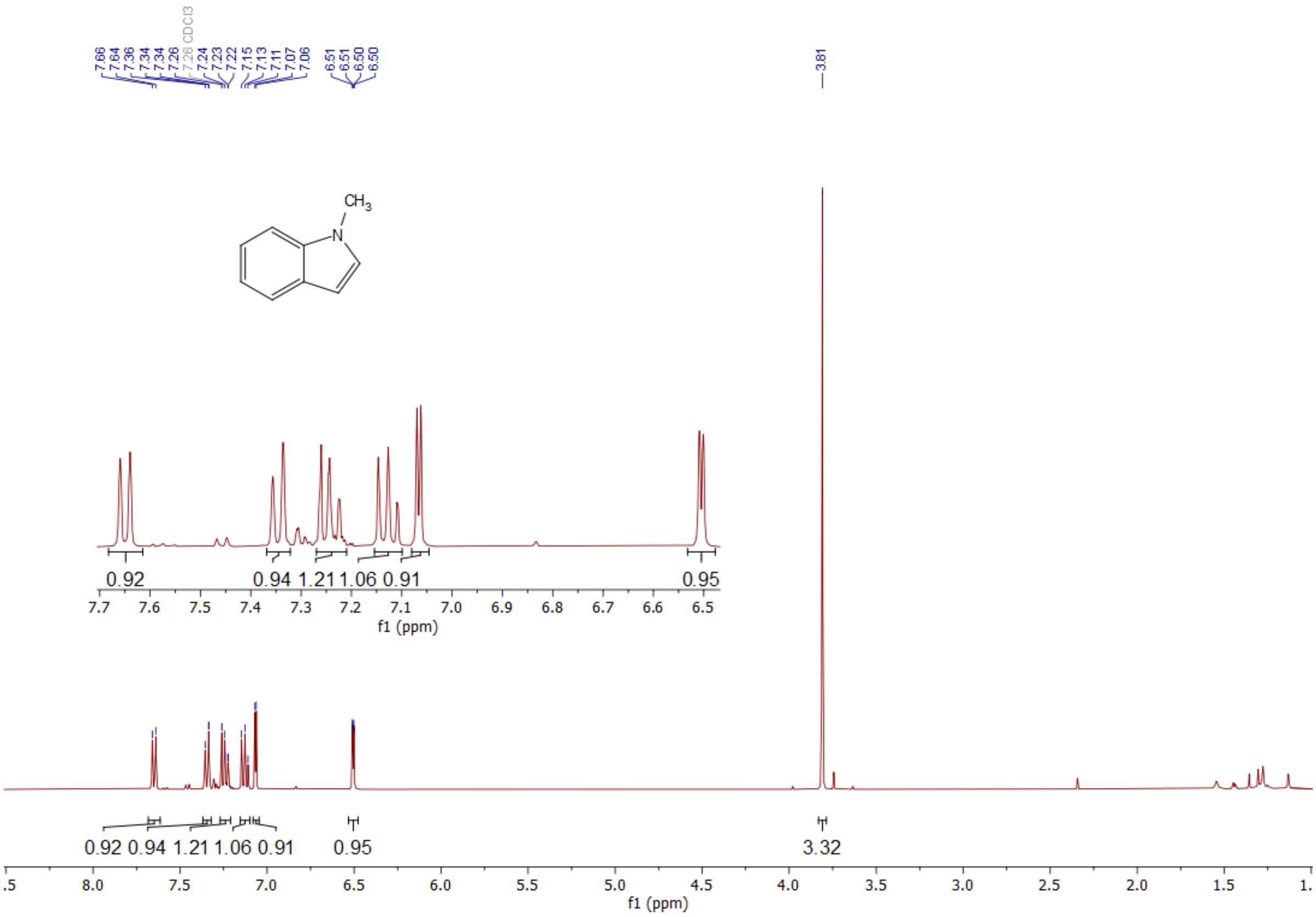
7.66
7.64
7.36
7.34
7.34
7.26 CDCl3
7.24
7.23
7.22
7.15
7.13
7.11
7.07
7.06
6.51
6.51
6.50
6.50
3.81
CH3
N
0.92
0.94 1.21 1.06 0.91
0.95
f1 (ppm)
0.92 0.94 1.21 1.06 0.91
0.95
3.32
f1 (ppm)
