# Supplementary material for: Dual screen for gut metabolites suppressing enterobacterial growth and invasiveness reveals structure–activity relationships among anti-infective indoles
Source: Gut Microbes. 2026 Sep 12;18(1):2728235. doi: 10.1080/19490976.2026.2728235 (PMC13577278; doi:10.1080/19490976.2026.2728235)
Supplement: Supplementary file 2 [file KGMI_A_2728235_SM2167.docx]

**Supplementary table S1.** Strains and plasmids used in this study

| Strain | Genotype | Reference |
| --- | --- | --- |
| *S*.Tm *wt* | SL1344, *wt* (SB300, Sm^R^) | (1) |
| *S*.Tm *ΔinvG* | SL1344, *ΔinvG* (SB161, Sm^R^) | (2) |
| *S*.Tm *ΔmotA* | SL1344, *ΔmotA* (SB300, Sm^R^) | (3) |
| *S*.Tm “wt” 14028 | 14028, *ΔmalX* (Kan^R^) | (4) |
| *S.fl* *wt* | M90T, *wt* | (5) |
| *S.fl* *ΔmxiD* | M90T, *ΔmxiD* (Kan^R^) | (6) |
| Plasmid | **Description** | **Reference** |
| pM975 | p*ssaG*-GFP (SPI2-dependent, vacuolar) | (7,8) |
| pZ1400 | p*uhpT*-GFP (cytosolic) | (9,10) |

**References for Supplementary table S1.**

1. Hoiseth SK, Stocker B a. D. Aromatic-dependent Salmonella typhimurium are non-virulent and effective as live vaccines. Nature. 1981 May;291(5812):238–9. doi:10.1038/291238a0

2. Kaniga K, Bossio JC, Galán JE. The Salmonella typhimurium invasion genes invF and invG encode homologues of the AraC and PulD family of proteins. Molecular Microbiology. 1994;13(4):555–68. doi:10.1111/j.1365-2958.1994.tb00450.x

3. Geiser P, Di Martino ML, Samperio Ventayol P, Eriksson J, Sima E, Al-Saffar AKh, et al. Salmonella enterica Serovar Typhimurium Exploits Cycling through Epithelial Cells To Colonize Human and Murine Enteroids. mBio. 2021 Jan 12;12(1):10.1128/mbio.02684-20. doi:10.1128/mbio.02684-20

4. Porwollik S, Santiviago CA, Cheng P, Long F, Desai P, Fredlund J, et al. Defined Single-Gene and Multi-Gene Deletion Mutant Collections in Salmonella enterica sv Typhimurium. PLOS ONE. 2014 Jul 9;9(7):e99820. doi:10.1371/journal.pone.0099820

5. Sansonetti PJ, Kopecko DJ, Formal SB. Involvement of a plasmid in the invasive ability of Shigella flexneri. Infect Immun. 1982 Mar;35(3):852–60. doi:10.1128/iai.35.3.852-860.1982 PubMed PMID: 6279518; PubMed Central PMCID: PMC351125.

6. Skovajsová E, Colonna B, Prosseda G, Sellin ME, Di Martino ML. The VirF21:VirF30 protein ratio is affected by temperature and impacts Shigella flexneri host cell invasion. FEMS Microbiol Lett. 2022 Feb 22;369(1):fnac043. doi:10.1093/femsle/fnac043

7. Hapfelmeier S, Stecher B, Barthel M, Kremer M, Müller AJ, Heikenwalder M, et al. The Salmonella Pathogenicity Island (SPI)-2 and SPI-1 Type III Secretion Systems Allow *Salmonella* Serovar *typhimurium* to Trigger Colitis via MyD88-Dependent and MyD88-Independent Mechanisms. The Journal of Immunology. 2005 Feb 1;174(3):1675–85. doi:10.4049/jimmunol.174.3.1675

8. Sellin ME, Müller AA, Felmy B, Dolowschiak T, Diard M, Tardivel A, et al. Epithelium-Intrinsic NAIP/NLRC4 Inflammasome Drives Infected Enterocyte Expulsion to Restrict *Salmonella* Replication in the Intestinal Mucosa. Cell Host & Microbe. 2014 Aug 13;16(2):237–48. doi:10.1016/j.chom.2014.07.001

9. Hausmann A, Böck D, Geiser P, Berthold DL, Fattinger SA, Furter M, et al. Intestinal epithelial NAIP/NLRC4 restricts systemic dissemination of the adapted pathogen *Salmonella* Typhimurium due to site-specific bacterial PAMP expression. Mucosal Immunology. 2020 May 1;13(3):530–44. doi:10.1038/s41385-019-0247-0

10. Geiser P, Di Martino ML, Lopes ACC, Bergholtz A, Sundbom M, Skogar M, et al. Determinants of divergent Salmonella and Shigella epithelial colonization strategies resolved in human enteroids and colonoids. mBio. 2025 Jul 9;16(7):e0091125. doi:10.1128/mbio.00911-25 PubMed PMID: 40444467; PubMed Central PMCID: PMC12239579.
