# Supplementary material for: Dual screen for gut metabolites suppressing enterobacterial growth and invasiveness reveals structure–activity relationships among anti-infective indoles
Source: Gut Microbes. 2026 Sep 12;18(1):2728235. doi: 10.1080/19490976.2026.2728235 (PMC13577278; doi:10.1080/19490976.2026.2728235)
Supplement: Supplementary file 3 [file KGMI_A_2728235_SM2174.docx]

**Supplementary table S4.** Primers used in this study

| Target | Forward sequence | Reverse sequence |
| --- | --- | --- |
| *invG* | ACCCGTAGTGCCCTTTTCTG | CATTCGCGTGTGGATTCGAC |
| *sipA* | AGATAGCCTGCCCATCGAAA | GGAAGTGGGTTTGTTGCGAA |
| *fliC* | CGTGTCAACCTGTGCCAAAG | CTAAACAAACTGGGTGGCGC |
| *fljB* | GTTCTGCGGGACCTGGTTAG | CTCTGATCTGGGTGCGGTAC |
| *recA* | TACCGAACATCACGCCAATC | GTATGATGAGCCAGGCGATG |
